# Comparison of Rifaximin Alone and With Quinolones in the Primary Prevention of Spontaneous Bacterial Peritonitis in Patients With Decompensated Chronic Liver Disease

**DOI:** 10.7759/cureus.55251

**Published:** 2024-02-29

**Authors:** Tahir Zaman, Muhammad Bilal Ahmed Attari, Adeel Ahmad, Muhammad Ahsan Butt, Khurram Fayyaz, Syeda Aeman Zubair

**Affiliations:** 1 Department of Medicine, Lahore General Hospital, Lahore, PAK; 2 Medicine, Chelsea and Westminster Hospital NHS Foundation Trust, Chelsea, GBR; 3 Department of Medicine, Azra Naheed Medical College, Lahore, PAK; 4 Department of Medicine, Liaquat National Hospital, Karachi, PAK

**Keywords:** chronic liver disease, cirrhosis, sbp, fluoroquinolone, rifaximin

## Abstract

Background

In cirrhotic patients with ascites, primary prevention of spontaneous bacterial peritonitis (SBP) is a key strategy to lower morbidity and death. Rifaximin and fluoroquinolone used alternately as main prophylaxis are as effective as reported. This study aimed to compare the frequency of occurrence of SBP in patients with decompensated chronic liver disease treated with rifaximin alone and in combination with fluoroquinolone.

Methodology

A total of 76 patients with hepatitis C virus-related decompensated chronic liver disease and ascites were divided into two groups based on matching age, sex, and Child-Pugh class. Group A (38 patients) received rifaximin 1,100 mg/day in two divided doses with daily fluoroquinolone 400 mg/day, whereas group B (38 patients) received rifaximin 1,100 mg/day alone as a two dosage. The patients were monitored for up to three months. The study’s endpoints were SBP, hepatocellular carcinoma, compliance failure, death, or liver transplantation.

Results

In this comparative study involving 76 patients, the demographic and clinical characteristics were assessed across two treatment groups: rifaximin alone (n = 38) and rifaximin with fluoroquinolone (n = 38). The combination therapy demonstrated a statistically significant reduction in SBP compared to rifaximin alone. Additionally, the overall survival rate was higher in the combination group. These findings suggest potential benefits of the combined approach in managing hepatic encephalopathy-related complications.

Conclusions

When compared to rifaximin alone for primary SBP prophylaxis, the combination of rifaximin with fluoroquinolone exhibited greater effectiveness with the same safety profile.

## Introduction

Up to 30% of individuals with cirrhosis may develop spontaneous bacterial peritonitis (SBP), a bacterial infection that is prevalent among patients with ascites, with a predicted 20% in-hospital mortality rate [1]. Those diagnosed with SBP may exhibit a broad range of symptoms, including localized symptoms and/or signs of peritonitis (e.g., vomiting, diarrhea, ileus), regardless of the presence of systemic inflammation (e.g., altered white blood cell count, tachycardia, tachypnea, shock), as well as new-onset deteriorating renal and liver function, and a form of hepatic encephalopathy [2]. At the other end of the range, nevertheless, are a sizable proportion of asymptomatic SBP patients. As a result, the diagnostic procedure is the primary basis for determining the presence of SBP [3].

An examination of ascitic fluid cells typically reveals a higher-than-normal number of neutrophils, with a count of at least 250/mm^3^ needed to confirm the diagnosis of SBP. The most prevalent positive infections in ascitic fluid cultures (which only occur in 40% of patients with proven SBP) are Gram-negative bacteria, mostly *Escherichia coli*, and Gram-positive cocci, primarily streptococci and enterococci [4]. Nevertheless, up to 60% of patients with elevated ascites neutrophil counts may have negative ascites cultures, a condition referred to as culture-negative SBP. Patients in a third group can have bactericides, a condition in which the ascitic neutrophil count is less than 250/mm^3^, yet the cultures are positive. Patients in all three groups should receive similar care because their medical conditions are comparable to those of classic SBP [5].

The main causes of enteric bacterial translocation from the lumen of the intestinal tract to mesenteric lymph nodes and other extraintestinal sites, which results in SBP, are decreased gastrointestinal motility and excess bacteria, which are frequently seen among people with liver cirrhosis [6,7]. Moreover, several humoral and cell-mediated immune system abnormalities increase the risk of infection with bacteria in cirrhotic patients who are at risk for SBP [8]. The mortality rate from SBP was over 90% when it was originally reported, but with prompt detection and medication, it is now only about 20%. Empirical antibiotic administration is a common treatment for SBP, which should be initiated as soon as the condition is diagnosed without waiting for the ascitic fluid culture findings [9,10]. The preferred medication is 3,6 cefotaxime, a third-generation cephalosporin with a suggested dose of 6 g/day for a minimum of five days as it has a high concentration of ascitic fluid and covers the majority of pathogenic microbes.

When administered intravenously for either seven days or two days and then five days orally, ciprofloxacin produces a similar SBP treatment rate and overall survival as that of cefotaxime [11].

An initial episode of SBP is very likely to occur in cirrhotic individuals with low ascitic fluid protein concentration (<1.5 g/dL) and/or elevated blood bilirubin levels. Additionally, it was shown that a year later, almost 70% of individuals who had survived an SBP episode would relapse. Several studies have assessed long-term prevention in individuals who have had SBP in the past or not [12]. The optimal preventive measure needs to be inexpensive, safe, and efficient in reducing the number of infectious agents from the stomach while maintaining beneficial flora (selective intestinal decontamination) [13].

Another broad-spectrum antibiotic that is absorbed from the intestines very slowly is rifaximin. It works by preventing the production of RNA in microorganisms. Rifaximin has been the subject of extensive research for its anti-diarrheal activity since the mid-1980s [14]. The US Food and Drug Administration approved it in 2004 to be used in the prevention of traveler’s diarrhea, and, in 2010, it was approved for hepatic encephalopathy. Rifaximin has shown broad-spectrum antibacterial activity toward aerobes and anaerobes, both Gram-positive and Gram-negative, with little possibility of causing resistant bacteria [15]. Quinolone antibiotics are a diverse class of bactericidal agents with a shared bicyclic core structure that is connected to 4-quinolone. They are used in the agriculture sector, particularly for the breeding of poultry, as well as in medicine for humans and animals to treat bacterial infections [16]. Fluoroquinolones, which have a fluorine atom in their chemical structure and are effective against both Gram-positive and Gram-negative bacteria, make up the majority of quinolone antibiotics now in use. Ciprofloxacin, one of the antibiotics most often used globally, is one example [17].

This study aimed to compare the frequency of occurrence of SBP in patients with decompensated chronic liver disease treated with rifaximin alone and in combination with fluoroquinolone.

## Materials and methods

**Figure 1:**
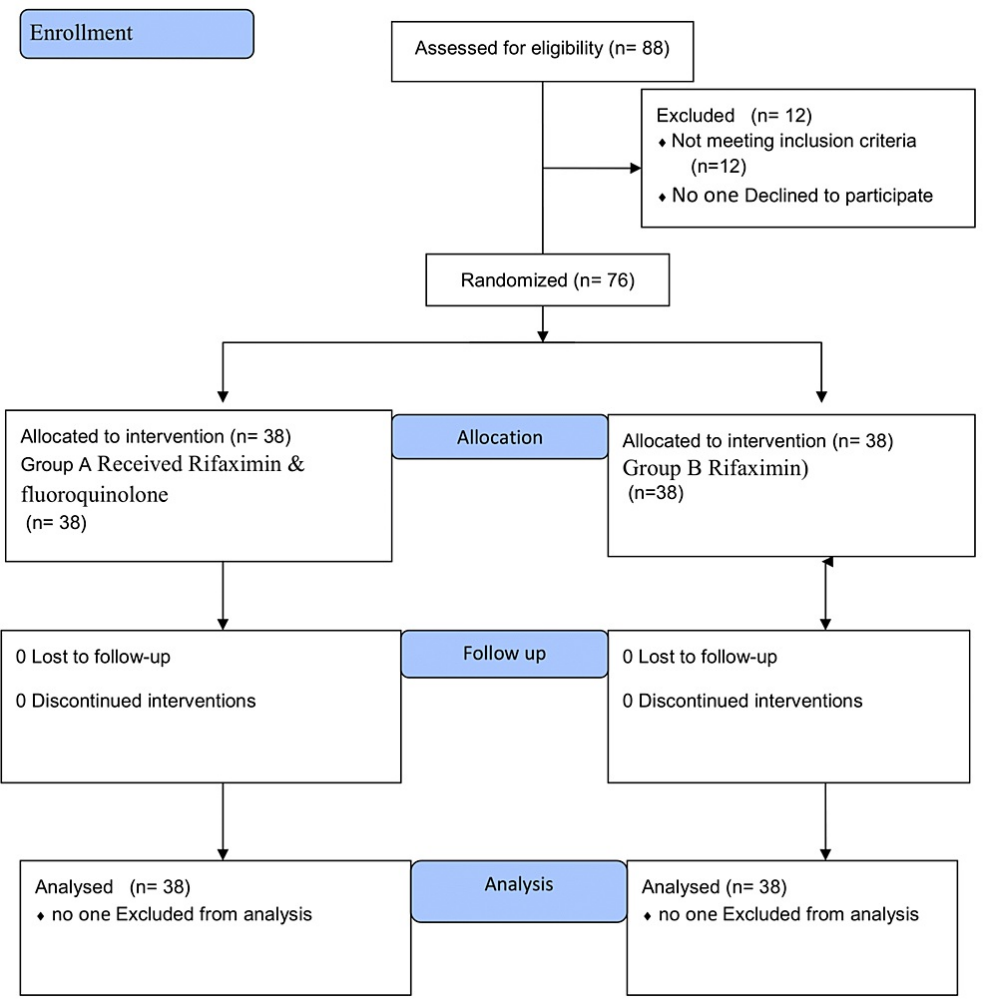
Consolidated Standards of Reporting Trials flowchart showing enrollment, intervention, allocation, and follow-up of the patients.

This single-blind randomized controlled trial was conducted in the Department of Medicine at Lahore General Hospital, a teaching hospital affiliated with Ameer-ud-Din Medical College in Pakistan. The ethical review board committee of Post Graduate Medical Institute/Ameer-ud-Din Medical College/Lahore General Hospital, Lahore, approved the study proposal (registration number: AMC/PGMI/LGH/00-177-20). The sample size was calculated using the Epi tool. The study by Mostafa et al. was used for the calculation of sample size, and the total sample size was calculated at 76 [18,19]. The study enrolled a total of 76 patients diagnosed with decompensated chronic liver disease and classified under Child-Pugh classes B and C. The patients were conveniently selected over three months from those presenting in the Department of Medicine, Lahore General Hospital. The sample was evenly divided into two groups, each comprising 38 patients. One group received rifaximin alone, while the other received a combination of norfloxacin and rifaximin. The sampling technique employed was convenient non-probability (non-random) to assess the comparative effectiveness of these treatments in managing liver cirrhosis. Figure 1 presents the Consolidated Standards of Reporting Trials flowchart showing patient enrollment, intervention, allocation, and follow-up.

Inclusion and exclusion criteria

Individuals meeting the following criteria were included: aged between 40 and 70 years, with a serum ascitic albumin gradient ≥1.1, ascitic fluid protein level <1.5 g/dL, and either severe liver disorders (Child-Pugh score ≥9 points, serum bilirubin ≥3 mg/dL) or kidney failure (serum creatinine ≥1.2 mg/dL, blood urea nitrogen ≥25 mg/dL, or salt level ≤130 mEq/L).

Patients with a prior history of SBP, prior antibiotic use within the previous two weeks, prior gastrointestinal bleeding within the last month, patients whose ascites resolved one month after diuretic therapy, uncontrolled diabetes mellitus, liver cancer, organic renal disease, human immunodeficiency virus infection, identified hypersensitivity to prescribed medications, enrolled in another study, or those who refused to provide consent were excluded.

Study groups (interventional group A and control group B)

Two groups of patients with similar ages, sexes, and Child-Pugh classes were formed. Group A (38 patients) received rifaximin 1,100 mg/day in two divided doses with daily fluoroquinolone 400 mg/day, whereas group B (38 patients) received rifaximin 1,100 mg/day alone as a two dosage.

Study protocol

The baseline assessment included ascitic fluid culture and analysis, liver and kidney tests, fresh urine sediment examination, and a comprehensive medical history. After independent randomization at each institution, medication commenced promptly, with follow-up appointments scheduled every four weeks. Patient compliance was assessed at each visit through discussions and pill counts. Non-compliance was defined as missing two or more appointments, taking less than 20% of prescribed tablets, or discontinuing the study medication for seven days or more. Medication discontinuation occurred in cases of upper gastrointestinal bleeding, liver transplant, or SBP episode (ascitic fluid neutrophil count ≥250 cells/mL with or without medical evidence). Monthly diagnostic paracentesis procedures were conducted or as required by the patient. Diagnosis of spontaneous bacteremia, SBP, urinary infections, and other pathogens was established through fluid cultures and analysis.

Data collection tool

A self-structured questionnaire was used to collect data after obtaining informed consent from the patients.

Data collection and procedure

A total of 76 consecutive patients with liver cirrhosis (Child-Pugh classes B and C) were enrolled in a three-month study at Lahore General Hospital. Antibiotic prophylaxis was administered to admitted patients following SBP episodes. Monthly complete blood count, liver function test, and prothrombin time/international normalized ratio tests were conducted, with re-evaluation after three months. After randomization into two groups (rifaximin alone vs. rifaximin with norfloxacin), patients were monitored for SBP, with ascitic taps and monthly follow-ups. Data on cirrhosis severity and ascitic fluid analysis were collected. Compliance, complications, and survival were tracked through regular appointments, investigating compliance failures, and conducting ascitic fluid trials for SBP diagnosis.

Outcomes and utilization

The development of SBP was the primary outcome of the study. The lower occurrence of SBP by one treatment modality relative to the other was provided by the medical practitioners with the option to offer better prophylaxis to patients with chronic liver disease.

Statistical analysis

Data recorded by the clinical performance was fed into SPSS software (IBM Corp., Armonk, NY, USA) and analyzed. Qualitative data were estimated through descriptive statistics. Quantitative data were expressed as mean ± standard deviation. For both groups, the percentage of patients developing SBP was considered and linked for both groups. A p-value of less than 0.05 was considered significant. The normality of the data was calculated. Non-parametric tests were applied and the Wilcoxon signed-rank test was applied to analyze the pre- and post-value difference within the groups. While the Mann-Whitney U test was employed to compare the pre- and post-value between groups.

Ethical considerations

The guidelines and procedures set by the ethical board of Lahore General Hospital/Post Graduate Medical Institute following the guidelines set by the University of Health Sciences, Lahore were followed, and the rights of research members were protected. Written informed consent was obtained from all participants. All information and data collected were kept confidential. Participants remained anonymous throughout the study. Participants were informed about the benefits and risks or disadvantages of the study procedure. They were also informed that they were free to withdraw at any time during the study. Data were kept confidential and password-protected.

## Results

In this study, we evaluated 76 patients. Table 1 presents the demographic and clinical characteristics of the two groups of patients: those treated with rifaximin alone (n = 38) and those treated with a combination of rifaximin and fluoroquinolone (n = 38). In the rifaximin group, patients had a mean age of 51.6 years, with 79.1% being men and 22.9% women. All patients were under primary prophylaxis. The distribution across Child-Pugh classes showed that 24.4% were in class B and 75.7% were in class C, yielding an average Child score of 12.51. Ascitis parameters included ascitic fluid protein at 0.8 ± 0.6 g/dL and serum albumin at 2.8 ± 0.4 g/dL. In the rifaximin and fluoroquinolone group, the average age was 54.7 years, with 76.1% being men and 23.9% women. All patients were under primary prophylaxis. The Child-Pugh class distribution showed 24.4% in class B and 75.7% in class C, yielding an average Child score of 12.47. Ascitic fluid protein was 1.2 ± 0.5 g/dL and serum albumin was 2.7 ± 0.5 g/dL.

**Table 1:** Comparison of the baseline values between the two groups.

Demographics and variables	Rifaximin (n = 38)	Rifaximin and fluoroquinolone (n = 38)
Age (years)	Mean ± standard deviation	Mean ± standard deviation
	51.6 ± 8.0	54.7 ± 7.5
Gender	Frequency (%)	Frequency (%)
Men	28 (79.1%)	25 (76.1%)
Women	10 (22.9%)	13 (23.9%)
Prophylaxis type	Frequency (%)	Frequency (%)
Primary	38 (100%)	38 (100%)
Child-Pugh class	Frequency (%)	Frequency (%)
B	10 (24.4%)	10 (24.4%)
C	28 (75.7%)	28 (75.7%)
Child score	Mean ± standard deviation	Mean ± standard deviation
	12.51 ± 2.05	12.47 ± 2.03
Ascites	Mean ± standard deviation	Mean ± standard deviation
Ascitic fluid protein (g/dL)	0.8 ± 0.6	1.2 ± 0.5
Serum albumin (g/dL)	2.8 ± 0.4	2.7 ± 0.5

Table 2 presents the key outcomes and comparisons between two treatment groups, one receiving rifaximin alone (n = 38) and the other a combination of rifaximin and fluoroquinolone (n = 4). The incidence of hepatic encephalopathy in the rifaximin group is 8.3%, while the combination group experiences it in 3.7% of cases. Regarding variceal bleeding, the new cases are 4.7% in the Rifaximin group and 16.11% in the combination group, with a statistically significant p-value of 0.007. The duration until the development of spontaneous bacterial peritonitis (SBP) is shorter in the Rifaximin group (median of 5.0 months) compared to the combination group (median of 9.50 months). Ascites culture results indicate a 16.7% positive rate in the Rifaximin group and a 0% rate in the combination group. Additionally, the table provides data on mortality, with an 8.3% death rate in the Rifaximin group and a 6.0% rate in the combination group.

**Table 2:** Contrast among the two groups related to follow-up trials. SBP: spontaneous bacterial peritonitis

	Rifaximin (n = 38)	Rifaximin and fluoroquinolone (n = 38)
Hepatic encephalopathy		
New	4 (8.3%)	2 (3.7%)
Hepatic encephalopathy		
New	6 (4.7%)	7 (16.11%)
Variceal bleeding	0.112	0.007
Months till SBP	5.0 (3.0–10.0)	9.50 (9.0–10.0)
Ascitis culture		
Positive	1/6 (16.7%)	0/2 (0%)
Negative	5/6 (83.3%)	2/2 (100%)
Death	4 (8.3%)	3 (6.0%)

Table 3 explains the key parameters related to treatment compliance and outcomes in the two cohorts receiving different therapeutic approaches. Compliance in both groups was 100%. The months until compliance failure revealed a notable difference between the two groups, with the rifaximin group having a mean duration of 9.0 ± 0.72 months, whereas the combination group had a shorter mean duration of 6.34 ± 1.28 months. Overall survival rates provided additional insights, indicating that 78.1% of patients in the rifaximin group and a higher proportion of 91.1% in the combination group survived. These findings underscore the potential impact of treatment compliance on patient outcomes and suggest that combination therapy may be associated with a higher overall survival rate.

**Table 3:** Comparison of overall survival and compliance.

	Rifaximin (n = 38)	Rifaximin and fluoroquinolone (n = 38)
Compliance	38 (100%)	38 (100%)
Months till compliance failure	9.0 ± 0.72	6.34 ± 1.28
Overall survival	34 (78.1%)	35 (91.1%)

Table 4 presents a statistical analysis of the two groups. The post and pre-treatment p-value for neutrophil levels in ascitic fluid was 0.160, indicating no significant difference in means between the groups. However, the post and pre-treatment bilirubin levels yielded a highly significant p-value of 0.00, suggesting a notable difference. Additionally, pre and post-treatment abdominal tenderness/pain showed a significant p-value of 0.008, signifying a distinct change in abdominal symptoms post-treatment. Notably, p-values for fever, vomiting, and constipation parameters are not provided.

**Table 4:** Wilcoxon signed-rank test for group A (rifaximin and fluoroquinolone) and group B (rifaximin).

Pre- and post-value of group A and group B		Group A (n = 38)		Group B (n = 38)	
Negative and positive ranks of pre and post-value of group A and group B		Mean rank	P-value	Mean rank	P-value
Post-level of neutrophil in ascitic fluid – Pre-level of neutrophil in ascitic fluid	Negative ranks	18.22	0.00	15.55	0.160
	Positive ranks	30.38		34.31	
Post-bilirubin level – Pre-bilirubin level	Negative ranks	20.19	0.00	15.92	0.00
	Positive ranks	7.00		7.00	
Post-abdominal tenderness/pain – pre-abdominal tenderness/pain	Negative ranks	0.00	0.46	0.00	0.008
	Positive ranks	2.50		4.00	
Post-fever – Pre-fever	Negative ranks	0.00	0.46	0.00	0.14
	Positive ranks	2.50		3.50	
Post-vomiting – Pre-vomiting	Negative ranks	0.00	0.46	0.00	0.14
	Positive ranks	2.50		3.50	
Post-constipation – Pre-constipation	Negative ranks	0.00	0.46	0.00	0.14
	Positive ranks	2.50		3.50	

Table 5 presents the findings of the Mann-Whitney U test employed to compare the distribution of ranks between two independent groups, i.e., group A and group B, across various parameters. The results are presented in the table, which includes the mean ranks, sum of ranks, and p-values for each comparison. Considering the parameter pre-level of neutrophil in ascitic fluid, the Mann-Whitney U test calculated a p-value of 0.917. This high p-value suggests that there is no statistically significant difference in the distribution of ranks between group A and group B for the pre-treatment levels of neutrophils in ascitic fluid. Contrastingly, for the post-level of neutrophil in ascitic fluid, the Mann-Whitney U test resulted in a p-value of 0.001, indicating a highly significant difference in the distribution of ranks between the two groups. This implies that the post-treatment levels of neutrophils in ascitic fluid differed significantly between group A and group B.

**Table 5:** Mann-Whitney U test for group A (rifaximin and fluoroquinolone) and group B (rifaximin).

Pre- and post-values	Groups	N	Mean rank	Sum of ranks	P-value
Pre-level of neutrophil in ascitic fluid	Group A	38	38.24	1,453.00	0.917
	Group B	38	38.76	1,473.00	
Post-level of neutrophil in ascitic fluid	Group A	38	29.63	1,126.00	0.001*
	Group B	38	47.37	1,800.00	
Pre-bilirubin level	Group A	38	39.08	1,485.00	0.819
	Group B	38	37.92	1,441.00	
Post-bilirubin level	Group A	38	36.74	1,396.00	0.483
	Group B	38	40.26	1,530.00	
Pre-abdominal tenderness/pain	Group A	38	38.50	1,463.00	1.000
	Group B	38	38.50	1,463.00	
Post-abdominal tenderness/pain	Group A	38	37.00	1,406.00	0.500
	Group B	38	40.00	1,520.00	
Pre-fever	Group A	38	38.50	1,463.00	1.000
	Group B	38	38.50	1,463.00	
Post-fever	Group A	38	37.50	1,425.00	0.500
	Group B	38	39.50	1,501.00	
Pre-vomiting	Group A	38	38.50	1,463.00	1.000
	Group B	38	38.50	1,463.00	
Post-vomiting	Group A	38	37.50	1,425.00	0.50
	Group B	38	39.50	1,501.00	
Pre-constipation	Group A	38	38.50	1,463.00	1.000
	Group B	38	38.50	1,463.00	
Post-constipation	Group A	38	37.50	1,425.00	0.50
	Group B	38	39.50	1,501.00	

## Discussion

In this comparative study involving 76 patients, the demographic and clinical characteristics were assessed across two treatment groups: rifaximin alone (n = 38) and rifaximin with fluoroquinolone (n = 38). The combination therapy demonstrated a statistically significant reduction in the development of SBP compared to rifaximin alone. Additionally, the overall SBP rate was lower in the combination group. These findings suggest potential benefits of the combined approach in managing liver cirrhosis-related complications. We have concluded that, when compared to rifaximin alone, primary SBP prophylaxis alternating between rifaximin with fluoroquinolone exhibited greater effectiveness with the same safety profile.

Rifaximin has a very good safety profile, as demonstrated by several clinical studies and post-marketing surveillance programs. This is likely because it has very little absorption from the stomach and, as a result, has little systemic activity [20]. Flatulence and nausea were among the few gastrointestinal side effects that were recorded. On the other hand, norfloxacin is more prone to cause systemic adverse effects as it can be absorbed easily from the stomach. Psychological signs including pain, sleeplessness, dizziness, and tiredness as well as gastrointestinal effects such as nausea, vomiting, and diarrhoea are frequently experienced [21]. Life-threatening arrhythmias, tendinopathy, and aggravation of myasthenia gravis are a few more uncommon but dangerous adverse effects [22]. In this study, there was no discernible difference between the mild-to-moderate and well-tolerated adverse effects of the two medications. However, it was difficult to distinguish between the typical dyspeptic signs of the portal hypertension condition (induced by diarrhea, enteropathy, colopathy, and cholecystopathy) and the real adverse effects of the tested antimicrobials, especially rifaximin [23]. This problem was also seen in other trials evaluating rifaximin’s effectiveness in treating hepatic encephalopathy. One study clarified that the majority of the symptoms were caused by cirrhosis complications or disease progression and that the gastrointestinal adverse events, which were regularly recorded in the trials, were not unexpected given patients with advanced liver disease were participating [24]. In this 24-month study, rifaximin (550 mg, twice daily) exhibited a safe profile with adverse events similar to a prior trial. Long-term use showed no increased infection risk or antibiotic resistance. Importantly, rifaximin maintained low rates of hepatic encephalopathy-related hospitalizations (0.21), comparable to the original trial, and lower than the placebo group (0.72). This suggests that extended rifaximin treatment sustains reduced hospitalization rates without heightened adverse events, emphasizing its safety and efficacy for long-term use in hepatic encephalopathy patients [25].

Three patient populations have been determined by the European Association for the Study of the Liver as being at high risk for developing SBP: individuals with acute digestive problems, hemorrhage, those with low total protein levels in ascitic fluid, and no prior record of SBP. On the other hand, there was a significant increase in the baseline levels of serum bilirubin, prothrombin time, and Child-Pugh scores among our patients who experienced SBP during the trial.

The likelihood of experiencing SBP in 152 ascitic patients receiving prophylactic antimicrobial therapy with rifaximin instead of the systemically absorbed antibiotic ciprofloxacin was only assessed retrospectively in one recent study by Lutz et al. [26]. Following a four-week follow-up, the study found that individuals receiving systemic antibiotic prophylaxis (n = 17) had a significantly reduced rate of SBP, but patients receiving rifaximin (n = 27) and those not receiving preventive medication (n = 108) had identical SBP rates. The increased incidence of SBP episodes under rifaximin compared to fluoroquinolone was thought to be caused by rifaximin resistance or the possibility that infections in SBP patients could not exclusively come from direct bacterial translocation from the gut. One potential cause of SBP could involve bacterial infection associated with inadequate dental hygiene, which can only be addressed by an intravenous antibiotic [27]. This study, however, is constrained by its brief duration, small patient population, and omission of any discussion of the potential long-term systemic side effects of ciprofloxacin, which is known to be more readily absorbed orally (80-100% bioavailable) than our investigated antibiotic, norfloxacin, which have a limited the absorption rate of 30-40%.

However, there have not been many trials comparing rifaximin to a placebo for the prevention of SBP in cirrhosis patients. According to a cohort study by Hanouneh et al., cirrhotic patients with ascites and no history of SBP who took rifaximin had a higher transplant-free survival benefit than those who did not get antibacterial prophylaxis [28]. When compared to a placebo, rifaximin substantially decreased the polymorphonuclear leukocytic count in the ascitic fluid of cirrhotic patients with refractory ascites, improving their overall health. This was indicated by further prospective case-control research conducted by Danulescu et al. [29].

Furthermore, Vlachogiannakos et al. demonstrated that patients who took rifaximin had a significantly higher five-year cumulative probability of survival and a significantly reduced chance of getting hepatic encephalopathy, hepatorenal syndrome, SBP, and variceal bleeding when compared to matched controls who did not receive antibiotic prophylaxis [30]. By contrast, our findings showed that rifaximin-treated patients experienced fewer hepatic encephalopathy episodes (4.7% versus 9.3%, correspondingly) than norfloxacin-treated individuals. Furthermore, it was noted that rifaximin decreased the number of new v episodes relative to previous episodes by 11.6% as opposed to 4.7% for norfloxacin. In both cases, the variation was not statistically significant. Additionally, patient survival was equivalent in the rifaximin and norfloxacin groups, with corresponding rates of mortality.

This study has several strengths, including a well-defined patient population with a robust sample size (n = 76) and a clear demarcation between two treatment groups (rifaximin alone and rifaximin with fluoroquinolone). The detailed characterization of patient demographics, clinical features, and treatment outcomes provides a comprehensive understanding of the study cohorts. The utilization of statistical analyses, such as the Mann-Whitney U test and Wilcoxon signed-rank test, adds rigor to the interpretation of results. Additionally, the inclusion of relevant parameters, such as hepatic encephalopathy, variceal bleeding, and treatment compliance, enhances the clinical significance of the findings. Despite its strengths, this study has certain limitations. Our study’s primary limitation is that it was a randomized controlled trial with a small sample size rather than a randomized, placebo-controlled trial. Selection and observer bias cannot be eliminated in this kind of research. Though the placebo group was eligible for prophylaxis according to standards, ethical issues must be addressed regarding a randomized controlled study that withheld any sort of antimicrobial prophylaxis from them given the elevated incidence of SBP. The unequal distribution in the number of patients between the two treatment groups, particularly in Table 2, raises concerns about potential bias. The retrospective nature of the analysis may introduce inherent limitations associated with data accuracy and completeness. Furthermore, the absence of p-values for certain parameters in Table 4 limits the comprehensive understanding of the statistical significance of those outcomes. Additionally, the study lacks information on specific comorbidities and medications that might impact treatment outcomes, limiting the generalizability of findings to a broader population. Future prospective studies with larger and more balanced cohorts could address these limitations and further validate the study’s findings.

## Conclusions

Our findings demonstrated that rifaximin with fluoroquinolone used alternately as primary prevention of SBP produced better outcomes than rifaximin alone.
